# Supplementary material for: DNA methylation presents distinct binding sites for human transcription factors
Source: eLife. 2013 Sep 3;2:e00726. doi: 10.7554/eLife.00726 (PMC3762332; doi:10.7554/eLife.00726)
Supplement: Supplementary file 1. — (A) 154 CpG-containing motifs tested on our protein microarray. (B) List of transcription factors and cofactors available on our protein microarray. (C) Transcription factors and cofactors binding to methylated DNA motif(s). (D) KLF4 binding methylated 6-mers with CpG at the center position obtained by integrating KLF4 ChIP-Seq and methylome data in human H1 cell. (E) Information of loci (L1–L5) tested in Figure 4C–F: genome locations (hg18), sequences, ChIP PCR and bisulfite-sequencing primers. DOI: http://dx.doi.org/10.7554/eLife.00726.026 [file elife00726s001.docx]

**Supplementary file 1A. 154 CpG-containing motifs tested on our protein microarray.**

| **Motif ID** | **Sequence** | **Sequence with spacer and primer** | **Source** |
| --- | --- | --- | --- |
| M1 | TTTCGCGC | TTTCGCGCAAATTTCGCGCCCCTATAGTGAGTGCTATTA | TRANSFAC |
| M2 | TTTCCGGAAA | TTTCCGGAAAGGGTTTCCGGAAACCCTATAGTGAGTGCTATTA | TRANSFAC |
| M10 | TGCGTGAGAAGA | TGCGTGAGAAGAGGGTGCGTGAGAAGACCCTATAGTGAGTGCTATTA | TRANSFAC |
| M18 | GGGAAACCGAAAC | GGGAAACCGAAACAAAGGGAAACCGAAACCCCTATAGTGAGTGCTATTA | TRANSFAC |
| M21 | GACCACGTGGTC | GACCACGTGGTCAAAGACCACGTGGTCCCCTATAGTGAGTGCTATTA | TRANSFAC |
| M24 | CTGCAGCGAGCAACTGAGAATCCAAGAC | CTGCAGCGAGCAACTGAGAATCCAAGACAAACTGCAGCGAGCAACTGAGAATCCAAGACCCCTATAGTGAGTGCTATTA | TRANSFAC |
| M26 | CGGGCAAAGGCCA | CGGGCAAAGGCCAAAACGGGCAAAGGCCACCCTATAGTGAGTGCTATTA | TRANSFAC |
| M27 | CGGATGT | CGGATGTAAACGGATGTCCCTATAGTGAGTGCTATTA | TRANSFAC |
| M28 | CGCCTGCGGA | CGCCTGCGGAAAACGCCTGCGGACCCTATAGTGAGTGCTATTA | TRANSFAC |
| M30 | CCTCGGCCGCCCCCTCGCGGC | CCTCGGCCGCCCCCTCGCGGCAAACCTCGGCCGCCCCCTCGCGGCCCCTATAGTGAGTGCTATTA | TRANSFAC |
| M31 | CCCTCCCG | CCCTCCCGAAACCCTCCCGCCCTATAGTGAGTGCTATTA | TRANSFAC |
| M32 | CCCGTGACC | CCCGTGACCAAACCCGTGACCCCCTATAGTGAGTGCTATTA | TRANSFAC |
| M35 | CACGTG | CACGTGAAACACGTGCCCTATAGTGAGTGCTATTA | TRANSFAC |
| M41 | AGGGTCGCGTGAGTATAAAAGCCGGTTTTCGGGG | AGGGTCGCGTGAGTATAAAAGCCGGTTTTCGGGGAAAAGGGTCGCGTGAGTATAAAAGCCGGTTTTCGGGGCCCTATAGTGAGTGCTATTA | TRANSFAC |
| M46 | AGTAGAAGCTGGGCCCCAGGCGTGGCGCTT | AGTAGAAGCTGGGCCCCAGGCGTGGCGCTTAAAAGTAGAAGCTGGGCCCCAGGCGTGGCGCTTCCCTATAGTGAGTGCTATTA | TRANSFAC |
| M47 | CACATCTGGACAGATGTGGGCG | CACATCTGGACAGATGTGGGCGAAACACATCTGGACAGATGTGGGCGCCCTATAGTGAGTGCTATTA | TRANSFAC |
| M48 | GCGCTTGCGCATGCG | GCGCTTGCGCATGCGAAAGCGCTTGCGCATGCGCCCTATAGTGAGTGCTATTA | TRANSFAC |
| M51 | CTGATTTCCCCGAAATGACGG | CTGATTTCCCCGAAATGACGGAAACTGATTTCCCCGAAATGACGGCCCTATAGTGAGTGCTATTA | TRANSFAC |
| M57 | TCTCACGCAA | TCTCACGCAACCCTCTCACGCAACCCTATAGTGAGTGCTATTA | TRANSFAC |
| M59 | GAAACCCCTGGAATATTCCCGAC | GAAACCCCTGGAATATTCCCGACAAAGAAACCCCTGGAATATTCCCGACCCCTATAGTGAGTGCTATTA | TRANSFAC |
| M60 | CCGGAAGT | CCGGAAGTCCCCCGGAAGTCCCTATAGTGAGTGCTATTA | TRANSFAC |
| M61 | CGCCTGCGC | CGCCTGCGCAAACGCCTGCGCCCCTATAGTGAGTGCTATTA | (*11*) |
| M62 | CGCATGCGC | CGCATGCGCAAACGCATGCGCCCCTATAGTGAGTGCTATTA | (*11*) |
| M63 | CGCACGCGC | CGCACGCGCAAACGCACGCGCCCCTATAGTGAGTGCTATTA | (*11*) |
| M65 | CCGCCGG | CCGCCGGAAACCGCCGGCCCTATAGTGAGTGCTATTA | (*11*) |
| M67 | CTCCGCCC | CTCCGCCCAAACTCCGCCCCCCTATAGTGAGTGCTATTA | (*11*) |
| M69 | CGTCACTTC | CGTCACTTCAAACGTCACTTCCCCTATAGTGAGTGCTATTA | (*11*) |
| M70 | CTTCCGC | CTTCCGCAAACTTCCGCCCCTATAGTGAGTGCTATTA | (*11*) |
| M71 | AGGGCGG | AGGGCGGAAAAGGGCGGCCCTATAGTGAGTGCTATTA | (*11*) |
| M73 | CGGCCCG | CGGCCCGAAACGGCCCGCCCTATAGTGAGTGCTATTA | (*11*) |
| M74 | AAGATGGCG | AAGATGGCGAAAAAGATGGCGCCCTATAGTGAGTGCTATTA | (*11*) |
| M75 | CGCGCAC | CGCGCACAAACGCGCACCCCTATAGTGAGTGCTATTA | (*11*) |
| M77 | CCGGGCC | CCGGGCCAAACCGGGCCCCCTATAGTGAGTGCTATTA | (*11*) |
| M78 | ATGGCGGC | ATGGCGGCAAAATGGCGGCCCCTATAGTGAGTGCTATTA | (*11*) |
| M80 | CCGGCCG | CCGGCCGAAACCGGCCGCCCTATAGTGAGTGCTATTA | (*11*) |
| M82 | TTCCGCC | TTCCGCCAAATTCCGCCCCCTATAGTGAGTGCTATTA | (*11*) |
| M83 | GCGCTCC | GCGCTCCAAAGCGCTCCCCCTATAGTGAGTGCTATTA | (*11*) |
| M85 | CTCCCCG | CTCCCCGAAACTCCCCGCCCTATAGTGAGTGCTATTA | (*11*) |
| M86 | CGCAGCC | CGCAGCCAAACGCAGCCCCCTATAGTGAGTGCTATTA | (*11*) |
| M89 | GCCACGCC | GCCACGCCAAAGCCACGCCCCCTATAGTGAGTGCTATTA | (*11*) |
| M95 | CTGCCGC | CTGCCGCAAACTGCCGCCCCTATAGTGAGTGCTATTA | (*11*) |
| M96 | AAATGGCG | AAATGGCGAAAAAATGGCGCCCTATAGTGAGTGCTATTA | (*11*) |
| M99 | CCGGCTC | CCGGCTCAAACCGGCTCCCCTATAGTGAGTGCTATTA | (*11*) |
| M116 | CGTAATTAGGAAGGTAAATC | CGTAATTAGGAAGGTAAATCCCCCGTAATTAGGAAGGTAAATCCCCTATAGTGAGTGCTATTA | (*12*) |
| M134 | AACTCCCATTAGCGTTAATGG | AACTCCCATTAGCGTTAATGGCCCAACTCCCATTAGCGTTAATGGCCCTATAGTGAGTGCTATTA | (*12*) |
| M137 | TCAGCACCACGGACAG | TCAGCACCACGGACAGAAATCAGCACCACGGACAGCCCTATAGTGAGTGCTATTA | (*12*) |
| M197 | ACTACATTTCCCG | ACTACATTTCCCGCCCACTACATTTCCCGCCCTATAGTGAGTGCTATTA | (*13*) |
| M199 | TCTCGCGAGA | TCTCGCGAGAAAATCTCGCGAGACCCTATAGTGAGTGCTATTA | (*13*) |
| M203 | TGACGTCA | TGACGTCAAAATGACGTCACCCTATAGTGAGTGCTATTA | (*13*) |
| M205 | GGCGCTCGCTGGTGACG | GGCGCTCGCTGGTGACGAAAGGCGCTCGCTGGTGACGCCCTATAGTGAGTGCTATTA | (*13*) |
| M206 | GTGACGT | GTGACGTAAAGTGACGTCCCTATAGTGAGTGCTATTA | (*13*) |
| M207 | TGCGCACG | TGCGCACGAAATGCGCACGCCCTATAGTGAGTGCTATTA | (*13*) |
| M209 | TGGATTGTCGCCAG | TGGATTGTCGCCAGAAATGGATTGTCGCCAGCCCTATAGTGAGTGCTATTA | (*13*) |
| M212 | GCGCGCGCG | GCGCGCGCGAAAGCGCGCGCGCCCTATAGTGAGTGCTATTA | (*13*) |
| M213 | GTCACGCCC | GTCACGCCCAAAGTCACGCCCCCCTATAGTGAGTGCTATTA | (*13*) |
| M223 | GGAAATCCCGC | GGAAATCCCGCAAAGGAAATCCCGCCCCTATAGTGAGTGCTATTA | (*13*) |
| M226 | GTTGCCGGGCAAC | GTTGCCGGGCAACAAAGTTGCCGGGCAACCCCTATAGTGAGTGCTATTA | (*13*) |
| M236 | TTTCCCGCC | TTTCCCGCCAAATTTCCCGCCCCCTATAGTGAGTGCTATTA | (*13*) |
| M237 | CGTCACG | CGTCACGAAACGTCACGCCCTATAGTGAGTGCTATTA | (*13*) |
| M239 | AGGAGCTCGAGTCC | AGGAGCTCGAGTCCAAAAGGAGCTCGAGTCCCCCTATAGTGAGTGCTATTA | (*13*) |
| M245 | CCGCCTCTTCCG | CCGCCTCTTCCGAAACCGCCTCTTCCGCCCTATAGTGAGTGCTATTA | (*13*) |
| M248 | CTTTCGATTT | CTTTCGATTTCCCCTTTCGATTTCCCTATAGTGAGTGCTATTA | (*13*) |
| M249 | CGCCATGGCAAC | CGCCATGGCAACAAACGCCATGGCAACCCCTATAGTGAGTGCTATTA | (*13*) |
| M251 | CCCGGAAAG | CCCGGAAAGAAACCCGGAAAGCCCTATAGTGAGTGCTATTA | (*14*) |
| M252 | ACTTGCGCC | ACTTGCGCCAAAACTTGCGCCCCCTATAGTGAGTGCTATTA | (*14*) |
| M253 | ACGTGATCT | ACGTGATCTCCCACGTGATCTCCCTATAGTGAGTGCTATTA | (*14*) |
| M254 | CCGCCCCCC | CCGCCCCCCAAACCGCCCCCCCCCTATAGTGAGTGCTATTA | (*14*) |
| M256 | GCGTCTTAC | GCGTCTTACAAAGCGTCTTACCCCTATAGTGAGTGCTATTA | (*14*) |
| M257 | GAACGCTAG | GAACGCTAGAAAGAACGCTAGCCCTATAGTGAGTGCTATTA | (*14*) |
| M259 | GCGCCAATG | GCGCCAATGAAAGCGCCAATGCCCTATAGTGAGTGCTATTA | (*14*) |
| M260 | ACTTCCGTT | ACTTCCGTTCCCACTTCCGTTCCCTATAGTGAGTGCTATTA | (*14*) |
| M261 | TAGCTACGA | TAGCTACGACCCTAGCTACGACCCTATAGTGAGTGCTATTA | (*14*) |
| M262 | CCCGTACTG | CCCGTACTGAAACCCGTACTGCCCTATAGTGAGTGCTATTA | (*14*) |
| M263 | CCGAAGACG | CCGAAGACGAAACCGAAGACGCCCTATAGTGAGTGCTATTA | (*14*) |
| M265 | CCGAGAACC | CCGAGAACCAAACCGAGAACCCCCTATAGTGAGTGCTATTA | (*14*) |
| M269 | GACGACGAA | GACGACGAAAAAGACGACGAACCCTATAGTGAGTGCTATTA | (*14*) |
| M270 | GCTCGAAAG | GCTCGAAAGAAAGCTCGAAAGCCCTATAGTGAGTGCTATTA | (*14*) |
| M271 | GCAACGGAG | GCAACGGAGAAAGCAACGGAGCCCTATAGTGAGTGCTATTA | (*14*) |
| M272 | TAGCGCAAT | TAGCGCAATCCCTAGCGCAATCCCTATAGTGAGTGCTATTA | (*14*) |
| M273 | AGCGAGTAT | AGCGAGTATCCCAGCGAGTATCCCTATAGTGAGTGCTATTA | (*14*) |
| M279 | CAAACGCTA | CAAACGCTACCCCAAACGCTACCCTATAGTGAGTGCTATTA | (*14*) |
| M282 | GCATGGCGG | GCATGGCGGAAAGCATGGCGGCCCTATAGTGAGTGCTATTA | (*14*) |
| M283 | TCGCTGTTG | TCGCTGTTGAAATCGCTGTTGCCCTATAGTGAGTGCTATTA | (*14*) |
| M284 | TGCGCAACA | TGCGCAACAAAATGCGCAACACCCTATAGTGAGTGCTATTA | (*14*) |
| M288 | GGCCACGTC | GGCCACGTCAAAGGCCACGTCCCCTATAGTGAGTGCTATTA | (*14*) |
| M289 | AATCCGACT | AATCCGACTCCCAATCCGACTCCCTATAGTGAGTGCTATTA | (*14*) |
| M290 | GAGGCGC | GAGGCGCAAAGAGGCGCCCCTATAGTGAGTGCTATTA | (*11*) |
| M291 | AGCGGCG | AGCGGCGAAAAGCGGCGCCCTATAGTGAGTGCTATTA | (*11*) |
| M292 | GGCGCCC | GGCGCCCTTTGGCGCCCCCCTATAGTGAGTGCTATTA | (*11*) |
| M293 | TCGCCGC | TCGCCGCAAATCGCCGCCCCTATAGTGAGTGCTATTA | (*11*) |
| M295 | CTGCGGC | CTGCGGCAAACTGCGGCCCCTATAGTGAGTGCTATTA | (*11*) |
| M296 | AGGCGCG | AGGCGCGTTTAGGCGCGCCCTATAGTGAGTGCTATTA | (*11*) |
| M297 | CCGCTGC | CCGCTGCAAACCGCTGCCCCTATAGTGAGTGCTATTA | (*11*) |
| M298 | CGGCTCC | CGGCTCCTTTCGGCTCCCCCTATAGTGAGTGCTATTA | (*11*) |
| M299 | AGCCCCG | AGCCCCGTTTAGCCCCGCCCTATAGTGAGTGCTATTA | (*11*) |
| M300 | CCCGCTC | CCCGCTCAAACCCGCTCCCCTATAGTGAGTGCTATTA | (*11*) |
| M301 | CCGGAGC | CCGGAGCAAACCGGAGCCCCTATAGTGAGTGCTATTA | (*11*) |
| M302 | CCCGGAG | CCCGGAGAAACCCGGAGCCCTATAGTGAGTGCTATTA | (*11*) |
| M303 | ACGTCAG | ACGTCAGAAAACGTCAGCCCTATAGTGAGTGCTATTA | (*11*) |
| M304 | AGGCCGC | AGGCCGCTTTAGGCCGCCCCTATAGTGAGTGCTATTA | (*11*) |
| M305 | CGCTGCC | CGCTGCCAAACGCTGCCCCCTATAGTGAGTGCTATTA | (*11*) |
| M306 | CGGGAGC | CGGGAGCTTTCGGGAGCCCCTATAGTGAGTGCTATTA | (*11*) |
| M308 | CGGCAGC | CGGCAGCTTTCGGCAGCCCCTATAGTGAGTGCTATTA | (*11*) |
| M310 | CGGGCTC | CGGGCTCAAACGGGCTCCCCTATAGTGAGTGCTATTA | (*11*) |
| M311 | GCCTCGC | GCCTCGCAAAGCCTCGCCCCTATAGTGAGTGCTATTA | (*11*) |
| M312 | TGACGCA | TGACGCATTTTGACGCACCCTATAGTGAGTGCTATTA | (*11*) |
| M313 | ACCCGGA | ACCCGGATTTACCCGGACCCTATAGTGAGTGCTATTA | (*11*) |
| M314 | ACGCGGC | ACGCGGCTTTACGCGGCCCCTATAGTGAGTGCTATTA | (*11*) |
| M315 | TGGGCGC | TGGGCGCTTTTGGGCGCCCCTATAGTGAGTGCTATTA | (*11*) |
| M316 | CTCGCCC | CTCGCCCTTTCTCGCCCCCCTATAGTGAGTGCTATTA | (*11*) |
| M317 | ACCCCGC | ACCCCGCAAAACCCCGCCCCTATAGTGAGTGCTATTA | (*11*) |
| M318 | CCGGAAC | CCGGAACTTTCCGGAACCCCTATAGTGAGTGCTATTA | (*11*) |
| M319 | CGCTTCC | CGCTTCCAAACGCTTCCCCCTATAGTGAGTGCTATTA | (*11*) |
| M320 | CCGCTTC | CCGCTTCTTTCCGCTTCCCCTATAGTGAGTGCTATTA | (*11*) |
| M322 | CATCCGGG | CATCCGGGAAACATCCGGGCCCTATAGTGAGTGCTATTA | (*11*) |
| M327 | ACGTGGG | ACGTGGGTTTACGTGGGCCCTATAGTGAGTGCTATTA | (*11*) |
| M328 | CGTTTCCG | CGTTTCCGTTTCGTTTCCGCCCTATAGTGAGTGCTATTA | (*11*) |
| M369 | CCGGACATAACG | CCGGACATAACGAAACCGGACATAACGCCCTATAGTGAGTGCTATTA | (*13*) |
| M372 | TCAAGGACG | TCAAGGACGTTTTCAAGGACGCCCTATAGTGAGTGCTATTA | (*13*) |
| M380 | TTCCCGAGGG | TTCCCGAGGGTTTTTCCCGAGGGCCCTATAGTGAGTGCTATTA | (*13*) |
| M382 | TGACGGTACAG | TGACGGTACAGAAATGACGGTACAGCCCTATAGTGAGTGCTATTA | (*13*) |
| M388 | GCTGCCTCCGAAAGG | GCTGCCTCCGAAAGGTTTGCTGCCTCCGAAAGGCCCTATAGTGAGTGCTATTA | (*13*) |
| M390 | GGCCGTTCT | GGCCGTTCTTTTGGCCGTTCTCCCTATAGTGAGTGCTATTA | (*14*) |
| M391 | GAAAGGCGA | GAAAGGCGAAAAGAAAGGCGACCCTATAGTGAGTGCTATTA | (*14*) |
| M392 | GCAACGTGT | GCAACGTGTTTTGCAACGTGTCCCTATAGTGAGTGCTATTA | (*14*) |
| M399 | AGGTCGTGC | AGGTCGTGCAAAAGGTCGTGCCCCTATAGTGAGTGCTATTA | (*14*) |
| M400 | GTCGTCGGC | GTCGTCGGCTTTGTCGTCGGCCCCTATAGTGAGTGCTATTA | (*14*) |
| M402 | CTAGTCGCT | CTAGTCGCTAAACTAGTCGCTCCCTATAGTGAGTGCTATTA | (*14*) |
| M403 | AGCGGATAG | AGCGGATAGTTTAGCGGATAGCCCTATAGTGAGTGCTATTA | (*14*) |
| M408 | AGTTTCGAC | AGTTTCGACCCCAGTTTCGACCCCTATAGTGAGTGCTATTA | (*14*) |
| M412 | GCTTTTACG | GCTTTTACGGGGGCTTTTACGCCCTATAGTGAGTGCTATTA | (*14*) |
| M413 | AACGGGTT | AACGGGTTTTTAACGGGTTCCCTATAGTGAGTGCTATTA | (*1*) |
| M414 | ACGATTAG | ACGATTAGCCCACGATTAGCCCTATAGTGAGTGCTATTA | (*1*) |
| M415 | CGTTGATC | CGTTGATCTTTCGTTGATCCCCTATAGTGAGTGCTATTA | (*1*) |
| M418 | ATTGGACG | ATTGGACGTTTATTGGACGCCCTATAGTGAGTGCTATTA | (*1*) |
| M421 | ATCGTGTC | ATCGTGTCAAAATCGTGTCCCCTATAGTGAGTGCTATTA | (*1*) |
| M422 | GACGAGAA | GACGAGAAGGGGACGAGAACCCTATAGTGAGTGCTATTA | (*1*) |
| M423 | ACTTCGCT | ACTTCGCTTTTACTTCGCTCCCTATAGTGAGTGCTATTA | (*1*) |
| M426 | AACCGTCG | AACCGTCGAAAAACCGTCGCCCTATAGTGAGTGCTATTA | (*1*) |
| M427 | AGATATCG | AGATATCGGGGAGATATCGCCCTATAGTGAGTGCTATTA | (*1*) |
| M429 | AGGACGAT | AGGACGATAAAAGGACGATCCCTATAGTGAGTGCTATTA | (*1*) |
| M430 | CCCGATAG | CCCGATAGAAACCCGATAGCCCTATAGTGAGTGCTATTA | (*1*) |
| M432 | AGAGTCCG | AGAGTCCGTTTAGAGTCCGCCCTATAGTGAGTGCTATTA | (*1*) |
| M435 | CACGACGC | CACGACGCTTTCACGACGCCCCTATAGTGAGTGCTATTA | (*1*) |
| M437 | AAAACGTA | AAAACGTACCCAAAACGTACCCTATAGTGAGTGCTATTA | (*1*) |
| M442 | AAGCGGAC | AAGCGGACTTTAAGCGGACCCCTATAGTGAGTGCTATTA | (*1*) |
| M447 | TGTTCCGA | TGTTCCGATTTTGTTCCGACCCTATAGTGAGTGCTATTA | (*1*) |
| M448 | GCGTGGAA | GCGTGGAATTTGCGTGGAACCCTATAGTGAGTGCTATTA | (*1*) |
| M450 | GTATCGGA | GTATCGGAAAAGTATCGGACCCTATAGTGAGTGCTATTA | (*1*) |
| M451 | GGGTACGG | GGGTACGGAAAGGGTACGGCCCTATAGTGAGTGCTATTA | (*1*) |
| M452 | ACGTGCAG | ACGTGCAGTTTACGTGCAGCCCTATAGTGAGTGCTATTA | (*1*) |
| M453 | GCTCGGTA | GCTCGGTATTTGCTCGGTACCCTATAGTGAGTGCTATTA | (*1*) |
| M455 | ACGGAGTA | ACGGAGTAAAAACGGAGTACCCTATAGTGAGTGCTATTA | (*1*) |
| M460 | CGCATAAA | CGCATAAACCCCGCATAAACCCTATAGTGAGTGCTATTA | (*1*) |

**Supplementary file 1B. List of transcription factors and cofactors available on our protein microarray.**

| **Transcription factors** | | | | | | | | | |
| --- | --- | --- | --- | --- | --- | --- | --- | --- | --- |
| AATF | ADNP | AEBP2 | AFF4 | AHR | AIFM1 | ALX1 | AMOT | APLP2 | ARFGAP2 |
| ARGFX | ARID3A | ARID3B | ARNT | ARNT2 | ARNTL | ARNTL2 | ASCC1 | ASCL1 | ASCL2 |
| ASCL3 | ATF1 | ATF2 | ATF3 | ATF4 | ATF5 | ATF6 | ATF6B | ATF7 | ATG4B |
| ATMIN | ATOH1 | ATOH7 | ATRX | AHRR | AIRE | ALX4 | BACH1 | BAD | BAPX1 |
| BARHL1 | BARX1 | BARX2 | BATF | BATF2 | BATF3 | BAZ2B | BBX | BCL11A | BCL6 |
| BCL6B | BCLAF1 | BHLHB2 | BLOC1S1 | BLZF1 | BMI1 | BNC | BOD1L | BRD3 | BRD9 |
| BRF1 | BRPF1 | BRPF3 | BTF3 | BCL11B | C11orf13 | C13orf15 | C14orf106 | C16orf80 | C19orf25 |
| C21orf7 | CBFA2T2 | CBFA2T3 | CBFB | CCDC71 | CCDC88B | CCNC | CDKL5 | CDX2 | CEBPB |
| CEBPD | CEBPE | CEBPG | CEBPZ | CEP250 | CHD4 | CHRAC1 | CITED1 | CITED2 | CIZ1 |
| CLOCK | CNBP | CNOT3 | COBRA1 | CORO1A | CPSF4 | CREB1 | CREB3 | CREB3L1 | CREB3L2 |
| CREB3L3 | CREB3L4 | CREB5 | CREBL2 | CREM | CRX | CRY1 | CSDA | CTCF | CTNNB1 |
| CUX1 | CXXC1 | CBX2 | CDX1 | CDX4 | CEBPA | DAXX | DCP1A | DDIT3 | DDRGK1 |
| DDX20 | DEAF1 | DIDO1 | DLX1 | DLX3 | DLX4 | DLX5 | DLX6 | DMBX1 | DMRT1 |
| DMRT3 | DMRTC2 | DMTF1 | DNAJC2 | DPF1 | DPF2 | DR1 | DRAP1 | DTX1 | DTX2 |
| DUX3 | DZIP1 | DMRT2 | E2F2 | E2F3 | E2F4 | E2F7 | E2F8 | E2F1 | E2F5 |
| E4F1 | EBF1 | EBF4 | EDF1 | EGLN1 | EGR2 | EGR3 | EHF | EHZF | ELF1 |
| ELF2 | ELF3 | ELF4 | ELF5 | ELK1 | ELK3 | ELK4 | ELL2 | EMX1 | EN2 |
| ENC1 | ENO1 | EOMES | EP400 | EPAS1 | ERF | ERG | ESR1 | ESR2 | ESRRA |
| ESRRG | ESX1L | ETS1 | ETS2 | ETV1 | ETV3 | ETV4 | ETV5 | ETV7 | EVX1 |
| EYA2 | EZH2 | EBF2 | EVX2 | FAM189B | FEZF2 | FHL2 | FIZ1 | FKBP1A | FLI1 |
| FLJ10211 | FLJ10697 | FLJ11078 | FLJ14299 | FLJ20079 | FLJ23233 | FLJ23765 | FLJ31030 | FLJ31413 | FLJ32191 |
| FLJ36155 | FOS | FOSB | FOSL1 | FOSL2 | FOXA1 | FOXA2 | FOXA3 | FOXC2 | FOXF1 |
| FOXH1 | FOXI1 | FOXJ1 | FOXJ2 | FOXL1 | FOXL2 | FOXM1 | FOXN2 | FOXN3 | FOXO3 |
| FOXP1 | FOXP2 | FOXP3 | FOXP4 | FOXS1 | FUBP3 | FOXN1 | GABPA | GATA1 | GATA2 |
| GATA3 | GATA4 | GATA5 | GATAD1 | GATAD2A | GATAD2B | GATSL3 | GFI1 | GFI1B | GLI |
| GLI3 | GLI4 | GLIS2 | GLRX2 | GLYR1 | GMEB1 | GRHL1 | GRHL2 | GRHL3 | GRLF1 |
| GSC | GSCL | GSH_2 | GTF2A1 | GTF2A2 | GTF2B | GTF2E1 | GTF2E2 | GTF2F2 | GTF2H2 |
| GTF2H3 | GTF2H4 | GTF2I | GTF2IRD1 | GTF3C2 | GTF3C3 | GTF3C5 | GBX2 | HAND1 | HAND2 |
| HATH6 | HBP1 | HCFC2 | HCLS1 | HDAC8 | HDX | HEMK1 | HES1 | HES2 | HES4 |
| HES6 | HESX1 | HEXIM1 | HEY1 | HEY2 | HEYL | HHEX | HIC2 | HIF1A | HIF3A |
| HINFP | HIRA | HIST2H2AA3 | HIST2H2AC | HKR1 | HLF | HLX | HMBOX1 | HMG20A | HMGA1 |
| HMGB1 | HMGB4 | HMGN1 | HMGN2 | HMGN5 | HNF1B | HNF4A | HOMEZ | HOXA1 | HOXA10 |
| HOXA11 | HOXA5 | HOXA6 | HOXA7 | HOXB1 | HOXB13 | HOXB2 | HOXB5 | HOXB6 | HOXB7 |
| HOXB9 | HOXC10 | HOXC11 | HOXC4 | HOXC5 | HOXC6 | HOXC8 | HOXD1 | HOXD10 | HOXD3 |
| HOXD4 | HOXD8 | HOXD9 | HPCAL1 | HSF1 | HSF2 | HSF4 | HSFX1 | HSFY1 | HSFY2 |
| HTATIP2 | HUMPPA | HZF12 | HOXA2 | HOXC12 | HOXC9 | DLX2 | ID1 | ID2 | ID3 |
| IKZF1 | IKZF2 | IKZF3 | IKZF4 | IKZF5 | ILF2 | ILF3 | ING1 | IPF1 | IRF1 |
| IRF2 | IRF3 | IRF4 | IRF5 | IRF6 | IRF8 | IRF9 | IRX2 | IRX3 | IRX4 |
| IRX6 | ISL1 | ISL2 | IVNS1ABP | JARID1A | JARID1C | JARID1D | JDP2 | JMJ | JMJD2C |
| JUN | JUNB | KAT2B | KBTBD10 | KBTBD5 | KBTBD7 | KCNH6 | KCNIP2 | KCNIP4 | KDM3B |
| KDM4D | KHDRBS1 | KIAA0296 | KIAA0478 | KIAA1190 | KIAA1441 | KLF1 | KLF10 | KLF11 | KLF12 |
| KLF15 | KLF3 | KLF4 | KLF5 | KLF7 | KLF8 | KLHDC5 | KLHL1 | KLHL12 | KLHL13 |
| KLHL14 | KLHL29 | KLHL3 | KLHL36 | KLHL4 | KLHL6 | KLHL8 | KRBA1 | L3MBTL | L3MBTL4 |
| LARP1 | LAS1L | CERS4 | LBX2 | LCORL | LDOC1 | LEF1 | LHX2 | LHX4 | LHX5 |
| LHX6 | LITAF | LMO1 | LMO4 | LMX1A | LMX1B | ZNF827 | ZNF800 | LSR | LYL1 |
| LZTR1 | LZTS1 | LBX1H | MADH7 | MADH9 | MAFB | MAFF | MAFG | MAGEA4 | MAGED4 |
| MAGEF1 | MAX | MAZ | MECOM | MED1 | MED21 | MED27 | MED30 | MED4 | MEF2A |
| MEF2D | MEIS1 | MEIS2 | MEIS3 | MEOX1 | MEOX2 | MESP1 | MET | METTL3 | ZNF524 |
| MID1 | MID2 | MIER1 | MIER2 | MIER3 | MITF | MKX | MLLT3 | MLLT6 | MLLT7 |
| MLX | MLXIPL | MNAT1 | MNDA | MNT | MSC | MSX1 | MSX2 | MTA1 | MTA2 |
| MTA3 | MTF1 | MTPAP | MXD1 | MXD3 | MXD4 | MXI1 | MYB | MYBL2 | MYC |
| MYCL1 | MYCN | MYEF2 | MYF6 | MYNN | MYOD1 | MYOG | MYST2 | MYT1 | MZF1 |
| MLLT1 | NACC2 | NCALD | NCOA3 | NEUROD1 | NEUROD2 | NEUROD4 | NEUROD6 | NEUROG2 | NEUROG3 |
| NFAT5 | NFATC1 | NFATC3 | NFATC4 | NFE2 | NFE2L1 | NFE2L2 | NFE2L3 | NFIA | NFIB |
| NFIC | NFIL3 | NFIX | NFKB1 | NFYA | NFYB | NFYC | NHLH1 | NHLH2 | NKRF |
| NKX2-1 | NKX2-3 | NKX2-5 | NKX2-8 | NKX2_2 | NKX3_1 | NKX6_2 | NME1 | NME2 | NPAS1 |
| NPAS2 | NPM1 | NR0B1 | NR0B2 | NR1D1 | NR1D2 | NR1H2 | NR1H3 | NR1I2 | NR1I3 |
| NR2C1 | NR2C2 | NR2E1 | NR2E3 | NR2F1 | NR2F2 | NR2F6 | NR3C1 | NR3C2 | NR4A1 |
| NR4A2 | NR5A1 | NR5A2 | NR6A1 | NRBF2 | NRF1 | NUCB1 | NUFIP1 | NANOG | NFATC2 |
| NR1H4 | OLIG1 | OLIG2 | OLIG3 | ONECUT2 | OTP | OTUD4 | OTX1 | OTX2 | OVOL1 |
| OVOL2 | ONECUT1 | PATZ1 | PAX1 | PAX3 | PAX4 | PAX5 | PAX6 | PAX7 | PAX8 |
| PAX9 | PAXIP1 | PBF | PBRM1 | PBX2 | PCGF3 | PDLIM5 | PDS5B | PER1 | PGBD1 |
| PHF15 | PHF16 | PHF17 | PHF21A | PHOX2A | PHTF1 | PIAS1 | PIAS3 | PIM3 | PIR |
| PITX1 | PITX2 | PITX3 | PKNOX1 | PKNOX2 | PLAG1 | PLAGL1 | PLAGL2 | PMS1 | POGZ |
| POLE3 | POLR3K | POU1F1 | POU2F1 | POU2F2 | POU2F3 | POU3F2 | POU3F3 | POU4F3 | POU5F1 |
| POU6F1 | POU6F2 | PPARA | PPARD | PPARG | PPIE | PPP1R13L | PQBP1 | PRDM1 | PRDM10 |
| PRDM14 | PRDM15 | PRDM4 | PRDM5 | PRDM9 | PREB | PRKCBP1 | PRKRIP1 | PRKRIR | PROP1 |
| PRR7 | PRRX1 | PRRX2 | PSIP1 | PURA | PURG | PBX4 | PRDM16 | RAB18 | RAN |
| RARA | RARB | RARG | RAX2 | RBBP5 | RBM4 | RBM4B | RBM9 | RBMY1J | RBPJ |
| RBPJL | RCAN1 | RCVRN | REL | RELA | RELB | RFPL3 | RFWD3 | RFX1 | RFX2 |
| RFX3 | RFX4 | RFX5 | RFX6 | RFXANK | RHOXF1 | RING1 | RLIM | RNF110 | RNF112 |
| RNF113B | RNF114 | RNF115 | RNF135 | RNF138 | RNF141 | RNF2 | RNF4 | RORA | RORB |
| RORC | RUFY3 | RUNX1T1 | RUNX3 | RXRA | RXRB | RXRG | RYBP | REPIN1 | SAFB |
| SALL2 | SALL3 | SALL4 | SAP30 | SAP30BP | SATB2 | SCAND1 | SCAND2 | SCAPER | SCMH1 |
| SCML1 | SCML2 | SCML4 | SEMA4A | SETBP1 | SFMBT1 | SFRS7 | SHOX | SHOX2 | SIM2 |
| SIX1 | SIX2 | SIX5 | SLC30A9 | SMAD1 | SMAD2 | SMAD3 | SMAD4 | SMAD5 | SMARCA3 |
| SMARCA4 | SMARCB1 | SMARCC1 | SMARCC2 | SMARCE1 | SNAI1 | SNAI2 | SNAI3 | SNAPC2 | SNAPC4 |
| SNAPC5 | SND1 | SNF8 | SNRNP27 | SOX10 | SOX12 | SOX13 | SOX15 | SOX2 | SOX21 |
| SOX30 | SOX5 | SOX7 | SOX8 | SOX9 | SP100 | SP110 | SP140L | SP2 | SPDEF |
| SPI1 | SPIB | SPIC | SREBF1 | SREBF2 | SRF | SRY | SSBP2 | SSBP3 | SSX2 |
| SSX3 | SSX4 | ST18 | STAT1 | STAT2 | STAT3 | STAT4 | STAT5A | STAT5B | STAT6 |
| SUB1 | SUMO1 | SUPT4H1 | SUPT5H | SOLH | T | TADA2A | TADA3 | TAF1 | TAF10 |
| TAF11 | TAF13 | TAF1A | TAF1B | TAF5L | TAF6 | TAF7 | TAF7L | TAF9 | TARP |
| TBP | TBPL1 | TBR1 | TBX19 | TBX2 | TBX20 | TBX21 | TBX22 | TBX3 | TBX5 |
| TBX6 | TCEAL1 | TCEAL2 | TCERG1 | TCF12 | TCF19 | TCF21 | TCF3 | TCF4 | TCF7 |
| TCF7L2 | TCF8 | TCFL5 | TEAD1 | TEAD2 | TEAD3 | TEAD4 | TECPR1 | TEF | TERF1 |
| TERF2 | TET3 | TFAM | TFAP2A | TFAP2B | TFAP2C | TFAP2D | TFAP2E | TFAP4 | TFB2M |
| TFCP2 | TFCP2L1 | TFDP1 | TFDP2 | TFE3 | TFEB | TFEC | TFPT | TGIF1 | TGIF2 |
| TGIF2LX | THAP3 | THAP4 | THAP5 | THRA | THRB | TIAL1 | TIMELESS | TLX2 | TLX3 |
| TMEM175 | TMIGD2 | TOX | TOX2 | TOX4 | TP53 | TP73 | TRIM13 | TRIM16 | TRIM22 |
| TRIM24 | TRIM25 | TRIM26 | TRIM28 | TRIM31 | TRIM32 | TRIM34 | TRIM40 | TRIM69 | TRIP4 |
| TRMT1 | TSC22D1 | TSC22D3 | TSC22D4 | TSNAX | TTF2 | TULP1 | TULP4 | TWIST1 | TWIST2 |
| TAL1 | TAL2 | TCF15 | TLX1 | TRERF1 | UBE2K | UBP1 | UNKL | USF1 | USF2 |
| VAX1 | VAX2 | VDR | VENTX | VPS4B | VPS72 | VSX2 | WBP11 | WHSC1 | WT1 |
| WIZ | XBP1 | YAF2 | YBX1 | YBX2 | YEATS4 | YY1 | ZBED1 | ZBED2 | ZBED5 |
| ZBTB1 | ZBTB11 | ZBTB12 | ZBTB16 | ZBTB2 | ZBTB20 | ZBTB22 | ZBTB25 | ZBTB26 | ZBTB3 |
| ZBTB32 | ZBTB33 | ZBTB34 | ZBTB37 | ZBTB39 | ZBTB4 | ZBTB43 | ZBTB44 | ZBTB46 | ZBTB48 |
| ZBTB49 | ZBTB5 | ZBTB6 | ZBTB7A | ZBTB7B | ZBTB9 | ZCCHC11 | ZCCHC13 | ZCCHC14 | ZCCHC2 |
| ZCCHC3 | ZCCHC6 | ZCCHC7 | ZCCHC8 | ZCCHC9 | ZCRB1 | ZDHHC1 | ZEB2 | ZFAND3 | ZFAND5 |
| ZFD25 | ZFP1 | ZFP161 | ZFP2 | ZFP28 | ZFP3 | ZFP30 | ZFP36 | ZFP36L2 | ZFP37 |
| ZFP42 | ZFP64 | ZFP82 | ZFP90 | ZFP91 | ZFPL1 | ZHX1 | ZHX2 | ZHX3 | ZIC3 |
| ZIC4 | ZIM2 | ZIM3 | ZKSCAN1 | ZKSCAN3 | ZKSCAN5 | ZMYM3 | ZMYM5 | ZMYM6 | ZMYND11 |
| ZNF10 | ZNF124 | ZNF131 | ZNF132 | ZNF133 | ZNF134 | ZNF136 | ZNF137 | ZNF138 | ZNF140 |
| ZNF143 | ZNF146 | ZNF155 | ZNF157 | ZNF16 | ZNF160 | ZNF165 | ZNF167 | ZNF169 | ZNF174 |
| ZNF175 | ZNF177 | ZNF18 | ZNF180 | ZNF182 | ZNF184 | ZNF187 | ZNF189 | ZNF19 | ZNF192 |
| ZNF193 | ZNF2 | ZNF20 | ZNF200 | ZNF202 | ZNF205 | ZNF207 | ZNF213 | ZNF215 | ZNF217 |
| ZNF219 | ZNF22 | ZNF221 | ZNF222 | ZNF223 | ZNF224 | ZNF225 | ZNF226 | ZNF228 | ZNF23 |
| ZNF230 | ZNF232 | ZNF238 | ZNF239 | ZNF24 | ZNF248 | ZNF25 | ZNF250 | ZNF253 | ZNF256 |
| ZNF257 | ZNF259 | ZNF26 | ZNF263 | ZNF264 | ZNF266 | ZNF267 | ZNF271 | ZNF273 | ZNF274 |
| ZNF277 | ZNF28 | ZNF280B | ZNF280D | ZNF281 | ZNF287 | ZNF295 | ZNF296 | ZNF3 | ZNF300 |
| ZNF302 | ZNF304 | ZNF317 | ZNF319 | ZNF32 | ZNF322A | ZNF323 | ZNF324 | ZNF326 | ZNF331 |
| ZNF333 | ZNF334 | ZNF34 | ZNF343 | ZNF345 | ZNF35 | ZNF350 | ZNF354A | ZNF354C | ZNF358 |
| ZNF366 | ZNF37A | ZNF383 | ZNF384 | ZNF385A | ZNF385B | ZNF385D | ZNF394 | ZNF396 | ZNF398 |
| ZNF408 | ZNF41 | ZNF410 | ZNF414 | ZNF415 | ZNF416 | ZNF426 | ZNF432 | ZNF433 | ZNF434 |
| ZNF436 | ZNF44 | ZNF440 | ZNF443 | ZNF444 | ZNF445 | ZNF446 | ZNF449 | ZNF45 | ZNF452 |
| ZNF454 | ZNF461 | ZNF462 | ZNF467 | ZNF468 | ZNF471 | ZNF473 | ZNF48 | ZNF480 | ZNF483 |
| ZNF484 | ZNF485 | ZNF486 | ZNF490 | ZNF491 | ZNF493 | ZNF496 | ZNF497 | ZNF498 | ZNF501 |
| ZNF502 | ZNF503 | ZNF507 | ZNF510 | ZNF511 | ZNF512B | ZNF513 | ZNF514 | ZNF518A | ZNF519 |
| ZNF526 | ZNF528 | ZNF529 | ZNF530 | ZNF544 | ZNF547 | ZNF548 | ZNF549 | ZNF550 | ZNF551 |
| ZNF554 | ZNF555 | ZNF556 | ZNF557 | ZNF558 | ZNF559 | ZNF560 | ZNF563 | ZNF564 | ZNF565 |
| ZNF566 | ZNF567 | ZNF57 | ZNF571 | ZNF573 | ZNF574 | ZNF576 | ZNF577 | ZNF581 | ZNF582 |
| ZNF584 | ZNF585B | ZNF587 | ZNF592 | ZNF593 | ZNF596 | ZNF597 | ZNF599 | ZNF606 | ZNF607 |
| ZNF608 | ZNF610 | ZNF613 | ZNF614 | ZNF615 | ZNF621 | ZNF622 | ZNF623 | ZNF625 | ZNF626 |
| ZNF630 | ZNF639 | ZNF641 | ZNF643 | ZNF644 | ZNF649 | ZNF653 | ZNF655 | ZNF658 | ZNF664 |
| ZNF668 | ZNF669 | ZNF670 | ZNF671 | ZNF672 | ZNF673 | ZNF677 | ZNF680 | ZNF683 | ZNF684 |
| ZNF688 | ZNF689 | ZNF69 | ZNF691 | ZNF692 | ZNF695 | ZNF697 | ZNF7 | ZNF702P | ZNF706 |
| ZNF707 | ZNF709 | ZNF71 | ZNF710 | ZNF711 | ZNF720 | ZNF74 | ZNF75A | ZNF75D | ZNF76 |
| ZNF764 | ZNF765 | ZNF766 | ZNF77 | ZNF771 | ZNF773 | ZNF777 | ZNF784 | ZNF785 | ZNF789 |
| ZNF79 | ZNF791 | ZNF8 | ZNF808 | ZNF81 | ZNF821 | ZNF83 | ZNF830 | ZNF84 | ZNF85 |
| ZNF92 | ZNF93 | ZNRD1 | ZRANB2 | ZSCAN1 | ZSCAN12 | ZSCAN16 | ZSCAN18 | ZSCAN2 | ZSCAN20 |
| ZSCAN21 | ZSCAN22 | ZSCAN4 | ZSCAN5A | ZW10 | ZXDC | ZZZ3 | ZFP106 | ZFP212 | ZFP62 |
| E2F6 | FOXC1 | HOXA9 | GTF2F1 | ZNF114 | CNOT6 | FEV | ID4 | NFKBIL2 | OSR2 |
| TCEB2 |  |  |  |  |  |  |  |  |  |
| **Cofactors** | | | | | | | | | |
| ABT1 | ACAD8 | AES | AGFG2 | ANP32A | APBB1 | ATAD3B | ATN1 | AVEN | BANP |
| BCOR | BRF2 | BTBD1 | BTBD12 | BTBD14B | BTBD3 | BTBD5 | BTBD6 | BTG3 | C10orf10 |
| C19orf2 | C5orf41 | C7orf11 | CALCOCO1 | CAND1 | CCNE1 | CCNH | CCNL1 | CCNT1 | CD80 |
| CDCA7L | CDK5 | CHURC1 | CIAO1 | CITED4 | COPS2 | CRABP2 | CREG | CRIP1 | CRIP2 |
| CRK | CRY2 | CSRP1 | CSRP2 | CSRP3 | CTBP1 | CTR9 | DCAF7 | DGCR6L | DMAP1 |
| DNM2 | DRG1 | ELL | ELL3 | EPC1 | EYA1 | EYA3 | EYA4 | FHL1 | FHL3 |
| FKBP1B | FLII | HDAC4 | HSF2BP | HSGT1 | IFT172 | IL31RA | ITGB3BP | KEAP1 | KIAA0669 |
| LDB1 | LDB2 | LIMD1 | LMO2 | LMO3 | LMO6 | MAGEA12 | MAGEA2B | MAGEA3 | MAGEA6 |
| MAGEA8 | MAGEA9 | MAGEB1 | MAGEB2 | MAGEB3 | MAGEB4 | MAGEB6 | MAGEC2 | MAGED1 | MAGEE1 |
| MAGEH1 | MBTPS2 | MDFI | MED16 | MED17 | MED23 | MED24 | MED26 | MED28 | MED31 |
| MED6 | MED7 | MED8 | MEN1 | MLF1 | MYBBP1A | MYCBP | NAB1 | NAB2 | NBR1 |
| NCOA2 | NFATC2IP | NFKBIA | NFKBIZ | NKAP | NMI | NPDC1 | NRG1 | NRIP1 | NRIP3 |
| NCOA6 | NFKBIE | OCEL1 | OPTN | OTUD7B | P38IP | PAF1 | PASD1 | PAWR | PCBD1 |
| PCQAP | PDLIM4 | PFDN5 | PHB2 | PHF3 | PIASY | PMF1 | POLR3E | POU2AF1 | PRKAR1A |
| PSMC3 | PSMC5 | PTGER3 | PYGO2 | RBBP4 | RBBP9 | RCOR2 | RRN3 | SAP18 | SEC14L2 |
| SERTAD1 | SERTAD2 | SIAH1 | SIAH2 | SIN3B | SIRT1 | SIRT2 | SKI | SKIL | SLTM |
| SNIP1 | SNW1 | SPEG | SRA1 | SSRP1 | SSX2IP | SUFU | SUPT3H | SMARCA2 | TAF12 |
| TAF9B | TCEB1 | TCERG1L | TGFB1 | TGFB1I1 | THRSP | TLE1 | TLE2 | TLE3 | TLE6 |
| TNF | TP53TG1 | TRAK1 | TRIP10 | TRIP13 | TRIP6 | TSFM | TSG101 | TULP2 | TULP3 |
| VGLL1 | VGLL2 | VGLL3 | VHL | WBP5 | WDR61 | WWTR1 | YWHAH | ASH2L | CALR |
| BCL3 | FHL5 | HSBP1 | KAT5 | NCOA4 | PLRG1 | PSMC2 | RCOR | RCOR3 | TTRAP |

| **Supplementary file 1C. Transcription factors and cofactors binding to methylated DNA motif(s).** | | | | |  |
| --- | --- | --- | --- | --- | --- |
| **TF hit** | | **Binding motif(s)** | **Subfamily** | **Cancer gene** | **Development gene** |
| ARID3B | | /M303/M304/M318/M319/ | other | Suppressor | - |
| ARNT2 | | /M319/ | HLH | - | Development |
| ASH2L | | /M209/ | cofactor | - | Development |
| ATF6B | | /M298/M299/ | bZIP | - | - |
| BCL3 | | /M300/M301/M302/M303/M304/M305/M310/M311/M312/M313/M315/M316/M317/M318/M319/M320/M27/M322/M327/M31/M372/M447/M452/M67/M71/M74/M83/M85/M86/M89/M95/ | cofactor | oncogene | Development |
| CALR | | /M197/M209/M18/M213/ | cofactor | - | Development |
| CERS4 | | /M203/M279/M283/M24/ | Homeobox | - | - |
| CRX | | /M116/ | Homeobox | - | Development |
| DIDO1 | | /M24/ | Zinc finger | Suppressor | - |
| E2F3 | | /M212/M30/M41/M205/M63/M1/M245/M263/M291/M293/M134/M137/M60/M197/M2/M203/M206/M209/M18/M213/M236/M248/M249/M251/M252/M253/M257/M261/M262/M265/M272/M273/M279/M282/M283/M288/M24/M292/M295/M297/M300/M301/M302/M303/M304/M305/M306/M308/M311/M312/M313/M315/M316/M317/M318/M319/M320/M27/M322/M327/M31/M32/M372/M380/M391/M399/M403/M408/M35/M432/M442/M447/M448/M450/M452/M453/M455/M67/M69/M70/M71/M74/M77/M78/M82/M85/M86/M89/M95/M99/M47/M59/ | E2F TDP | - | - |
| E2F6 | | /M213/ | E2F TDP | - | - |
| FEZF2 | | /M197/M209/ | Zinc finger | - | Development |
| FOXC1 | | /M73/M10/ | Fork head | - | Development |
| GATA3 | | /M300/M304/M311/M313/M316/M318/M319/M327/M380/M388/M437/M452/M83/M86/M95/M99/ | Zinc finger | - | Development |
| GATA4 | | /M212/M30/M137/M60/M197/M2/M203/M206/M209/M18/M213/M236/M239/M252/M261/M265/M271/M273/M279/M282/M288/M24/M292/M300/M301/M302/M303/M304/M305/M306/M308/M310/M26/M311/M312/M313/M315/M316/M317/M318/M319/M320/M27/M322/M327/M31/M32/M372/M380/M388/M391/M399/M403/M408/M35/M413/M414/M415/M418/M421/M437/M442/M447/M448/M450/M451/M452/M453/M455/M67/M69/M70/M71/M74/M77/M78/M82/M83/M85/M86/M89/M95/M96/M99/M59/ | Zinc finger | - | Development |
| GTF2F1 | | /M203/ | other | - | - |
| HOXA5 | | /M41/M48/M1/M314/M28/M369/M400/M435/M61/M65/M73/M75/M51/M422/M57/M10/M116/M134/M137/M60/M197/M2/M203/M209/M18/M213/M292/M300/M301/M302/M303/M304/M305/M306/M308/M310/M26/M311/M312/M313/M315/M316/M317/M318/M319/M320/M27/M322/M327/M31/M32/M372/M380/M382/M388/M399/M403/M408/M35/M412/M413/M414/M415/M418/M421/M423/M427/M429/M430/M432/M437/M442/M447/M448/M450/M451/M452/M453/M455/M460/M67/M69/M70/M71/M74/M77/M78/M82/M83/M85/M86/M89/M95/M96/M99/M47/ | Homeobox | - | Development |
| HOXA9 | | /M413/M452/M74/ | Homeobox | oncogene | Development |
| KEAP1 | | /M413/M452/M96/ | cofactor | - | Development |
| KLF4 | | /M197/ | Zinc finger | Suppressor | Development |
| MEF2A | | /M203/M265/ | other | - | Development |
| NFATC1 | | /M2/ | other | - | - |
| NFIC | | /M209/ | other | - | - |
| NPAS2 | | /M99/ | HLH | - | Development |
| NRF1 | | /M30/M41/M205/M1/M291/M293/M213/M301/M305/M310/M313/M318/M319/M27/M322/M327/M32/M372/M380/M442/M447/M448/M450/M452/M455/M67/M69/M70/M71/M74/M78/M95/M96/ | other | - | Development |
| PHF21A | | /M203/ | Zinc finger | - | - |
| PMS1 | | /M203/M206/ | HMG box | Suppressor | - |
| PPARG | | /M212/M262/M271/M288/M24/M292/M297/M300/M301/M302/M303/M304/M305/M306/M308/M310/M26/M311/M312/M313/M315/M316/M317/M318/M319/M320/M27/M322/M327/M31/M32/M372/M380/M382/M388/M391/M399/M402/M403/M413/M414/M415/M427/M430/M432/M437/M442/M447/M448/M450/M452/M455/M67/M70/M71/M74/M78/M83/M85/M86/M89/M95/M96/M99/M47/M59/ | Zinc finger | - | Development |
| RBPJ | | /M265/ | other | Suppressor | Development |
| RNF138 | | /M400/M435/M61/M422/M213/M292/M300/M301/M302/M303/M304/M305/M306/M310/M26/M311/M312/M313/M315/M316/M317/M318/M319/M320/M27/M327/M31/M32/M372/M380/M382/M388/M399/M403/M35/M414/M432/M437/M442/M447/M448/M450/M451/M452/M453/M455/M67/M70/M74/M78/M82/M83/M85/M86/M89/M95/M96/M99/ | Zinc finger | - | - |
| RXRA | | /M30/M63/M1/M369/M435/M51/M18/M213/M288/M292/M300/M301/M302/M303/M304/M305/M306/M308/M310/M26/M311/M312/M313/M315/M316/M317/M318/M319/M320/M27/M322/M327/M31/M32/M372/M380/M388/M403/M414/M415/M442/M447/M448/M450/M451/M452/M453/M67/M70/M74/M78/M83/M85/M86/M89/M95/M96/ | Zinc finger | - | Development |
| SCAPER | | /M212/M30/M41/M1/M245/M291/M369/M400/M62/M65/M51/M137/M197/M2/M209/M18/M213/M262/M288/M24/M292/M300/M301/M302/M303/M304/M305/M306/M308/M310/M26/M311/M312/M313/M315/M316/M317/M318/M319/M320/M27/M322/M327/M31/M32/M372/M380/M382/M388/M390/M399/M403/M408/M35/M413/M414/M415/M418/M421/M423/M427/M430/M432/M437/M442/M447/M448/M450/M451/M452/M453/M455/M67/M69/M70/M71/M74/M77/M78/M82/M83/M85/M86/M89/M95/M96/M99/M47/M59/ | Zinc finger | - | - |
| SIRT1 | | /M304/ | cofactor | Suppressor | Development |
| SMAD4 | | /M300/M301/M302/M303/M304/M305/M311/M312/M313/M315/M316/M317/M318/M319/M320/M27/M322/M327/M31/M32/M380/M414/M447/M452/M95/ | other | Suppressor | Development |
| SSRP1 | | /M300/M304/M305/M312/M313/M315/M316/M317/M319/ | cofactor | - | - |
| SUB1 | | /M197/ | other | - | - |
| TFAP2A | | /M41/M1/M209/M18/M213/M249/M252/M261/M262/M265/M271/M272/M279/M282/M24/M292/M301/M302/M303/M306/M310/M319/M320/M27/M322/M327/M32/M380/M59/ | other | Suppressor | Development |
| TGIF1 | | /M269/M293/M328/M28/M369/M400/M426/M435/M62/M422/M116/M197/M392/M402/M403/M408/M35/M442/M453/ | Homeobox | - | Development |
| ZCCHC7 | | /M30/M213/M300/M301/M302/M303/M304/M305/M306/M310/M26/M311/M312/M315/M316/M317/M318/M319/M27/M322/M327/M32/M380/M399/M403/M408/M35/M413/M415/M437/M447/M448/M450/M455/M70/M71/M78/M86/M89/M95/M99/ | Zinc finger | - | - |
| ZKSCAN5 | | /M245/M269/M293/M369/M51/M57/M197/M209/M18/M257/M261/M262/M265/M270/M271/M272/M273/M279/M282/M283/M288/M382/M402/M408/ | Zinc finger | - | - |
| ZMYM3 | | /M203/M206/ | Zinc finger | - | Development |
| ZNF114 | | /M212/M30/M205/M48/M63/M1/M296/M314/M328/M369/M400/M426/M435/M62/M65/M73/M75/M51/M422/M57/M116/M137/M60/M197/M2/M203/M206/M209/M18/M213/M223/M226/M236/M239/M248/M249/M251/M252/M253/M256/M257/M259/M260/M21/M261/M262/M265/M270/M271/M272/M273/M279/M282/M283/M284/M288/M289/M290/M24/M292/M295/M297/M298/M299/M300/M301/M302/M303/M304/M305/M306/M308/M310/M26/M311/M312/M313/M315/M316/M317/M318/M319/M320/M27/M322/M327/M31/M32/M372/M380/M382/M388/M390/M391/M392/M399/M402/M403/M408/M35/M412/M413/M414/M415/M418/M421/M423/M427/M429/M430/M432/M437/M442/M447/M448/M450/M451/M452/M453/M455/M460/M67/M69/M70/M71/M74/M77/M78/M82/M83/M85/M86/M89/M95/M96/M99/M47/M59/ | Zinc finger | - | - |
| ZNF22 | | /M298/M299/ | Zinc finger | - | Development |
| ZNF28 | | /M452/M67/ | Zinc finger | - | - |
| ZNF416 | | /M212/M30/M41/M205/M48/M63/M1/M199/M245/M263/M269/M291/M293/M400/M435/M134/M137/M60/M197/M2/M203/M206/M209/M18/M213/M223/M226/M236/M239/M248/M249/M251/M252/M253/M256/M257/M259/M260/M21/M261/M262/M265/M270/M271/M272/M273/M279/M282/M283/M288/M289/M290/M24/M292/M295/M297/M300/M301/M302/M303/M304/M305/M306/M308/M310/M26/M311/M312/M313/M315/M316/M317/M318/M319/M320/M27/M322/M327/M31/M32/M372/M380/M382/M388/M390/M391/M392/M399/M402/M403/M408/M35/M412/M413/M414/M415/M418/M421/M423/M427/M429/M430/M432/M437/M442/M447/M448/M450/M451/M452/M453/M455/M460/M67/M69/M70/M71/M74/M77/M78/M82/M83/M85/M86/M89/M95/M96/M99/M47/M59/ | Zinc finger | - | - |
| ZNF461 | | /M369/M426/M435/M65/M422/M10/M116/ | Zinc finger | - | - |
| ZNF695 | | /M212/M41/M205/M1/M291/M137/M60/M197/M2/M203/M206/M209/M18/M213/M223/M226/M236/M239/M248/M249/M251/M252/M253/M256/M257/M259/M260/M21/M261/M262/M265/M270/M271/M272/M273/M279/M282/M283/M288/M289/M290/M24/M292/M295/M297/M300/M301/M302/M303/M304/M305/M306/M308/M310/M26/M311/M312/M313/M315/M316/M317/M318/M319/M320/M27/M322/M327/M31/M32/M372/M380/M382/M388/M390/M391/M392/M399/M402/M403/M408/M35/M59/ | Zinc finger | - | - |

| **Supplementary file 1D. KLF4 binding methylated 6-mers with CpG at the center position obtained by integrating KLF4 ChIP-Seq and methylome data in human H1 cell.** |
| --- |

| **6-mer** | **No. of 6-mer in foreground (summit only)** | | **No. of 6-mer in background** | | Enrichment | p-value |
| --- | --- | --- | --- | --- | --- | --- |
|  | **M ≥ 0.8** | **all M** | **M ≥ 0.8** | **all M** |  |  |
| CCCGGC | 1980 | 3980 | 4209 | 9302 | 1.10 | 0 |
| GCCGCC | 1140 | 3436 | 2361 | 8272 | 1.16 | 0 |
| CCCGCC | 2020 | 5169 | 4299 | 12120 | 1.10 | 2.48E-10 |
| CGCGCC | 599 | 2045 | 1206 | 4888 | 1.19 | 2.20E-08 |
| CACGCC | 2214 | 3114 | 4424 | 6564 | 1.05 | 9.21E-08 |
| CTCGGC | 1823 | 2906 | 3748 | 6378 | 1.07 | 2.95E-07 |
| GCCGCA | 935 | 1669 | 1947 | 3813 | 1.10 | 5.16E-06 |
| AGCGGC | 1018 | 2032 | 2045 | 4475 | 1.10 | 5.67E-06 |
| GGCGGA | 1274 | 2443 | 2640 | 5471 | 1.08 | 1.75E-05 |
| CCCGAG | 1393 | 2325 | 3113 | 5571 | 1.07 | 2.15E-05 |
| CTCGCC | 1062 | 2033 | 2201 | 4569 | 1.08 | 6.66E-05 |
| ACCGCG | 448 | 889 | 864 | 1946 | 1.14 | 8.95E-05 |
| CCCGGG | 955 | 1969 | 2097 | 4709 | 1.09 | 2.67E-04 |
| CCCGCA | 1219 | 2164 | 2542 | 4826 | 1.07 | 3.44E-04 |
| CGCGGC | 395 | 1786 | 831 | 4412 | 1.17 | 3.94E-04 |
| AGCGGG | 1241 | 2310 | 2593 | 5127 | 1.06 | 3.41E-03 |
| GACGGC | 780 | 1280 | 1587 | 2791 | 1.07 | 4.94E-03 |
| CCCGGA | 1030 | 2020 | 2162 | 4522 | 1.07 | 9.21E-03 |

**Supplementary file 1E. Information of loci (L1-L5) tested in Figures 4C-F: genome locations (hg18), sequences, ChIP PCR and bisulfite-sequencing primers.**

| L1 | chr1: 559311-559516 | | |
| --- | --- | --- | --- |
|  | CTAGCCCACTTCTTACCACAAGGCACACCTACACCCCTTATCCCTATACTAGTTATTATCGAAACCATCAGCCTACTCATTCAACCAATAGCCCTGGCCGTACGCCTAACCGCTAACATTACTGCAGGCCACCTACTCATGCACCTAATTGGAAGCGCCACACTAGCAATATCAACTATTAACCTTCCCTCTACACTTATCATCTT | | |
|  | ChIP PCR and qPCR primers | F | AGGCACACCTACACCCCTTA |
|  |  | R | AAGATGATAAGTGTAGAGGGAAGG |
|  | Bisulfite sequencing primers | F | TGGGTGTAGTGATTATAGGTTTTTG |
|  |  | R | TTTCTCATATTACATCACACCATCA |
| L2 | chr5:44424678-44424792 | | |
|  | CACCCACGTCCACCATTTACCGGGAGGCTCCAGAGGCGTAGGCAGCGGATCCGAGAAAGGAGCGAGGGGAGTCAGCCGGCTTTTCCGAGGAGTTATGGATGTTGGTGCATTCACT | | |
|  | ChIP PCR and qPCR primers | F | CACCCACGTCCACCATTTAC |
|  |  | R | AGTGAATGCACCAACATCCA |
|  | Bisulfite sequencing primers | F | GGAGTGAAGAGTGTTGGTGATT |
|  |  | R | TCAACACTAAAAATTATCTCATCAAAA |
| L3 | chr16:4681299-4681481 | | |
|  | TCATCTCAAGGCATTTGCTGAGCACCTGCTGTGCGTCAGTGGAGGCAGAAGAGCTGAGGCCCCGGCCCTGGCCCCGGGCGCCTGCCTTCCAGGCAGTGAGATGCCCAGCAGCTAATCAGAATCCTGCCAGGTTTGGTTACTATTAGGAAGAAAGTCAGCATGGTGGTGAGATGAACCCGTACA | | |
|  | ChIP PCR and qPCR primers | F | TCATCTCAAGGCATTTGCTG |
|  |  | R | TGTACGGGTTCATCTCACCA |
|  | Bisulfite sequencing primers | F | TTAGGGTTTTTAGGTTTGTGAGTAG |
|  |  | R | AACCCTCAAAACTATTTAAATCTCC |
| L4 | chr2:132747088-132747377 | | |
|  | TATTTAGCTGATTGTTGCTTTCATCTTCGGTAGTGGCGGTGAAAGAGGCATGACACTAAATCGACCCTTAGGACGCTCCCCTCCGTCCCCACCCCTCACCCCCTTCCCACACACACCCTCATTCCTGCACCCCCTCCTCAAACGCAAGAAAGGGAGAAAGACAGAAATTAAAGTAACAGGTGAGCCTCCAAGGCGGTGGAGGCGGGGGATCTCAAAGGGTGAGCAAGCGATGGGGGTCGGGGGATGTCTTGGCTGAGCTATCAAAAATAGGGGACCCACTTTCCAGCCCC | | |
|  | ChIP PCR and qPCR primers | F | CACACACACCCTCATTCCTG |
|  |  | R | CTGGAAAGTGGGTCCCCTA |
|  | Bisulfite sequencing primers | F | TGGTAGTGGTGGTGAAAGAGG |
|  |  | R | CAACCCCACCCAAAAACTAA |
| L5 | chr12:81828301-81828506 | | |
|  | AGGCTCCAACCCCACGGCAGTGCCTAGGGGGTGAATGTTTACAGCTACTGAAGCCCCAGTGGGCATGTTACAGTATGCTCCTTTAGTTTAGCCCTCTATA[GGCGGC]TTGTGTTAGCTCAATTAGACCCCCTTCCTTATCACAAGGACAGAGGGATTTCTGTATCCCAGGGTTTCTTACCTTGGTGTATCAGAAGAATCGGATTACA | | |
|  | ChIP PCR and qPCR primers | F | AGTGCCTAGGGGGTGAATGT |
|  |  | R | AACCCTGGGATACAGAAATCC |
|  | Bisulfite sequencing primers | F | TGGTAGTGTTTAGGGGGTGAA |
|  |  | R | AAACCCTAAAATACAAAAATCCCTCT |

References

1. S. Hu *et al.*, *Cell* **139**, 610 (Oct 30, 2009).

2. A. Bateman *et al.*, *Nucleic Acids Res* **32**, D138 (Jan 1, 2004).

3. S. Hu, Z. Xie, S. Blackshaw, J. Qian, H. Zhu, *Cold Spring Harb Protoc*, pdb prot5614 (May, 2011).

4. M. Klug, M. Rehli, *Epigenetics* **1**, 127 (Jul-Sep, 2006).

5. X. Chen *et al.*, *Cell* **133**, 1106 (Jun 13, 2008).

6. J. M. Shields, V. W. Yang, *Nucleic Acids Res* **26**, 796 (Feb 1, 1998).

7. X. S. Liu, D. L. Brutlag, J. S. Liu, *Nat Biotechnol* **20**, 835 (Aug, 2002).

8. A. Schuetz *et al.*, *Cell Mol Life Sci* **68**, 3121 (Sep, 2011).

9. R. Lister *et al.*, *Nature* **462**, 315 (Nov 19, 2009).

10. J. D. Nelson, O. Denisenko, K. Bomsztyk, *Nat Protoc* **1**, 179 (2006).

11. O. Elemento, S. Tavazoie, *Genome Biol* **6**, R18 (2005).

12. X. Xie *et al.*, *Proc Natl Acad Sci U S A* **104**, 7145 (Apr 24, 2007).

13. X. Xie *et al.*, *Nature* **434**, 338 (Mar 17, 2005).

14. O. Elemento, N. Slonim, S. Tavazoie, *Mol Cell* **28**, 337 (Oct 26, 2007).
